# Topological Pattern Recognition of Severe Alzheimer's Disease via Regularized Supervised Learning of EEG Complexity

**DOI:** 10.3389/fnins.2018.00685

**Published:** 2018-10-04

**Authors:** Miaolin Fan, Albert C. Yang, Jong-Ling Fuh, Chun-An Chou

**Affiliations:** ^1^Department of Mechanical and Industrial Engineering, Northeastern University, Boston, MA, United States; ^2^Division of Interdisciplinary Medicine and Biotechnology, Beth Israel Deaconess Medical Center, Harvard Medical School, Boston, MA, United States; ^3^Institute of Brain Sciences, National Yang-Ming University, Taipei, Taiwan; ^4^Neurological Institute, Taipei Veterans General Hospital, Taipei, Taiwan; ^5^School of Medicine, National Yang-Ming University, Taipei, Taiwan

**Keywords:** Alzheimer's disease, EEG, complexity analysis, pattern recognition, LASSO

## Abstract

Alzheimer's disease (AD) is a progressive brain disorder with gradual memory loss that correlates to cognitive deficits in the elderly population. Recent studies have shown the potentials of machine learning algorithms to identify biomarkers and functional brain activity patterns across various AD stages using electroencephalography (EEG). In this study, we aim to discover the altered spatio-temporal patterns of EEG complexity associated with AD pathology in different severity levels. We employed the multiscale entropy (MSE), a complexity measure of time series signals, as the biomarkers to characterize the nonlinear complexity at multiple temporal scales. Two regularized logistic regression methods were applied to extracted MSE features to capture the topographic pattern of MSEs of AD cohorts compared to healthy baseline. Furthermore, canonical correlation analysis was performed to evaluate the multivariate correlation between EEG complexity and cognitive dysfunction measured by the Neuropsychiatric Inventory scores. 123 participants were recruited and each participant was examined in three sessions (length = 10 seconds) to collect resting-state EEG signals. MSE features were extracted across 20 time scale factors with pre-determined parameters (*m* = 2, *r* = 0.15). The results showed that comparing to logistic regression model, the regularized learning methods performed better for discriminating severe AD cohort from normal control, very mild and mild cohorts (test accuracy ~ 80%), as well as for selecting significant biomarkers arcoss the brain regions. It was found that temporal and occipitoparietal brain regions were more discriminative in regard to classifying severe AD cohort vs. normal controls, but more diverse and distributed patterns of EEG complexity in the brain were exhibited across individuals in early stages of AD.

## 1. Introduction

Alzheimer's disease (AD) is a neurodegenerative disorder characterized by progressive loss of memory and cognitive dysfunctions. Despite of many efforts, the pathological mechanism of AD progression still remains unsettled. In recent decades, the emerging field of interdisciplinary studies between computational cognitive and data sciences has enabled data-driven knowledge discovery systems for investigating multivariate patterns based on large-scale, complex brain data. More specifically, advances of machine learning techniques have contributed to the clinical science by not only improving the automated diagnostic/predictive tools, but also enhancing the understanding of pathological mechanism underlying AD progression. In the past years, there were studies to demonstrate the capability of machine learning algorithms in addressing the sophisticated patterns using various brain data, e.g., electroencephalography (EEG) and magnetic resonance imaging (MRI). Trambaiolli et al. (2011) identified the bipolar peaks of EEG signals as biomarkers for differentiating AD, mild cognitive impairments (MCI) and early dementia patients. Casanova et al. (2011) found that most informative voxels in structural MRI data locate in the gray and white matter tissues, which can discriminate patients from cognitive normal subjects accurately using large-scale regularization. Other studies encouraged the utilization of an integrative EEG biomarkers derived from various sources in order to provide predictive models with diverse and comprehensive information (Poil et al., 2013; Triggiani et al., 2017).

Among modern neuroimaging modalities, EEG as a non-invasive, inexpensive technique has drawn extensive attentions for investigating nonlinear dynamics of neuronal brain functions. It was reported that AD progression can be characterized by the reduced complexity in EEG signals, which is hypothesized to be related to the loss of neurons and possible connectivity caused by pathological aging process. A recent and comprehensive review is refered to Dauwels et al. (2010). In this study, we used Multiscale Entropy (MSE) for estimating the nonlinear complexity of EEG signals across multiple temporal scales (Costa et al., 2002). Previous studies investigated MSE as a measure of complexity for understanding AD pathology using univariate (Escudero et al., 2006; Park et al., 2007) and multivariate EEG dynamics (Labate et al., 2013). It was reported that the decreased complexity in short-time scale and increased complexity in long-time scale distinguish AD patients from normal controls (Mizuno et al., 2010; Yang et al., 2013). A recent study (Azami et al., 2017) also indicated the potentials of the second-order MSE features for characterizing EEG changes with AD progression. Moreover, correlation was found between MSE features from various brain regions and multiple neuropsychiatric symptoms, particularly in temporal and occipitoparietal electrodes (Yang et al., 2013). Since the previous study only assessed the bivariate correlation, we extend to investigate the relationships in a multivariate feature space by applying canonical correlation analysis (CCA) (Hotelling, 1936). CCA is a multivariate technique that is capable to capture multiple causes and effects to further investigate the relationship between MSE and neuropsychiatric symptoms.

One of the most challenging tasks for understanding AD pathology is to characterize the biomarkers and associated patterns that differentiate different AD severity levels. Considering the pathological aging of the brain is a highly heterogeneous process, the generalizability in many existing research studies is limited by the small sample size, large individual variability, and high-dimensional data structure. While most state-of-the-art machine learning algorithms suffered from over-fitting data and produced poor generalized prediction results, regularized learning methods attempt to address this over-fitting issue by adding a regularization term (called *L*_1_-norm or *L*_2_-norm) to the cost function. Least Absolute Shrinkage and Selection Operator (LASSO) (Tibshirani, 1996) is a classic method that builds a regression model of correlating input variables (MSE features in this study) to the prediction outcome (severe AD or not) while posing a penalty on the number of non-zero coefficients of input variables (*L*_1_-norm feature selection). Later, Elastic net (ENet) was proposed by combining *L*_1_-norm and *L*_2_-norm for the purpose of addressing several drawbacks of LASSO, including the group effect among input variables in addition to feature selection. The flexibility and variability of regularization methods allow one to develop variants for specific purposes (Tibshirani et al., 2005; Bach, 2008). Specifically, the interpretability/stability of feature selection is desired for providing scientific insights, since consistent feature selection across different samples and individuals is more likely to suggest a meaningful pattern (Fan and Chou, 2016). Stability selection (Meinshausen and Bühlmann, 2010) is thus proposed based on the combination of feature selection method and repeated subsampling. For the cost of computational resources, the stability selection aims to provide a statistical control on the error rate of feature selection in a sparse dataset.

Based on the general concept of stability selection approach, the present study intends to provide a stability-based feature selection and identify important EEG biomarkers using the frequency of selection across multiple replicates in cross validation. The objective of our study is two fold. On one hand, we are interested in characterizing the functional brain activities with varying temporal scales that best discriminate severity levels of AD groups and normal controls based on EEG complexity. On the other hand, we aim to profile the topolographic map of EEG biomarkers for various AD severity and investigate the multivariate correlation patterns to cognitive dysfunctions.

## 2. Materials and methods

### 2.1. Participants

One hundred and twenty-three participants were recruited from the Dementia Clinic at the Neurological Institute, Taipei Veterans General Hospital in Taiwan. The diagnosis for AD was based on the criteria of the National Institute of Neurological and Communicative Disorders and the Stroke/Alzheimer's Disease and Related Disorders Association (McKhann et al., 1984). All patients had received neurological examinations, laboratory tests, EEG monitoring, and neuroimaging evaluation during the diagnostic process. Our study was approved by the Institutional Review Board of Taipei Veterans General Hospital to conduct retrospective analysis of the patients' clinical and EEG data. We excluded patients who had other conditions that caused secondary dementia, such as vascular dementia, Parkinson's disease, hypothyroidism, vitamin B12 deficiency, syphilis, and prior history of major psychiatric illness (e.g., major depression, bipolar disorder, or schizophrenia). The participants were categorized into four groups according to their severity of dementia, assessed by the Clinical Dementia Rating (CDR) scale (Morris, 1993). In the following sections, we refer to these groups as HC (healthy control; *N* = 15), AD1 (very mild, CDR = 0.5; *N* = 15), AD2 (mild, CDR = 1; *N* = 69), and AD3 (moderate to severe, CDR = 2; *N* = 24).

### 2.2. EEG data acquisition and pre-processing

A routine EEG recordings were performed on all participants (Nicolet EEG, Natus Medical, Incorporated, San Carlos, CA, USA) in the EEG examination room at the Neurological Institute of Taipei Veterans General Hospital. The EEG recording protocol began with a 5-min habituation to the examining environment, followed by three consecutive sessions of 10–20 s with the eyes closed and then open, and a session of photo stimulation, while only the eye closed data was used in the present study. The recordings were performed using the international 10–20 system of 19 electrodes (Fp1, Fp2, F7, F3, Fz, F4, F8, T3, C3, Cz, C4, T4, T5, P3, Pz, P4, T6, O1, and O2) with linked ear reference, 256 Hz sampling rate and filtered at 0.05 Hz high-pass, 70 hZ low-pass and notch filter of 60 Hz, and impedance below 3 kΩ. Vigilance was monitored by the EEG technician, who alerted patients when signs of drowsiness appeared in the tracings. Vertical eyeball movement was detected from electrodes placed above and below the right eye, while the horizontal eyeball movement was detected from electrodes placed at the left outer canthus. EEG signals were preprocessed to remove the linear trend and visually inspected to ensure there were no eye movement artifacts. The EEG signals were exported in European Data Format and were processed using MATLAB 2016b (Mathworks, Inc.).

### 2.3. Multiscale entropy analysis (MSE)

In this study, we employed MSE (Costa et al., 2002) to measure the nonlinear complexity of EEG signal. Let us consider a single-channel EEG signals with length = *N*, denoted by {*x*_1_, *x*_2_, .…*x*_*N*_}. MSE provides an estimate of the sample entropy over multiple time scales in two steps: (1) the construction of coarse-grained time series based on various scale factors, denoted by τ, and (2) the estimation of sample entropy for each time scale. In the first step, the range of τ need to be pre-defined as a set of increasing integers starting from 1. (i.e., [1, 2, …T]). For each possible value of τ, the corresponding coarse-grained time series *y*_*j*_(τ) is obtained by applying a non-overlapping sliding window with length = τ and taking the average of all values in each window, represented by the following equation (1 ≤ *j* ≤ *N*/τ):

(1) $$\begin{array}{l} y_{j}(\tau )=\frac{1}{\tau }\sum_{i=(j-1)\tau +1}^{j\tau }x_{i}. \end{array}$$

If we denote *M* as the largest integer such that *M* ≤ *N*/τ, the coarse-grained time series is then rewritten as {*y*_1_(τ), *y*_2_(τ), …, *y*_*j*_(τ), …*y*_*M*_(τ)}.

In the second step, the sample entropy (Richman and Moorman, 2000) is calculated for each coarse-grained time series as a function of τ. To calculate the sample entropy for a time series with length = *M*, two parameters need to be determined: the pattern length *m* and the similarity criterion *r*. Within the coarse-grained time series {*y*_1_(τ), *y*_2_(τ), …, *y*_*j*_(τ), …*y*_*M*_(τ)}, we denote a vector of pattern length = *m* as *Y*_*m*_(*k*) = {*y*_*k*_(τ), *y*_*k*+1_(τ), …, *y*_*k*+*m*−1_(τ)}. Accordingly, the total number of pairs of vectors that satisfy *D*(*Y*_*m*_(*k*), *Y*_*m*_(*l*)) < *r*(*k*≠*l*) is denoted by *N*_*m*_. The sample entropy *I*(τ) for this time series with parameters τ and *r* is defined as:

(2) $$\begin{array}{r} I\left(\tau ,r\right)=-\log\frac{N_{m+1}}{N_{m}}. \end{array}$$

**Figure 1:**
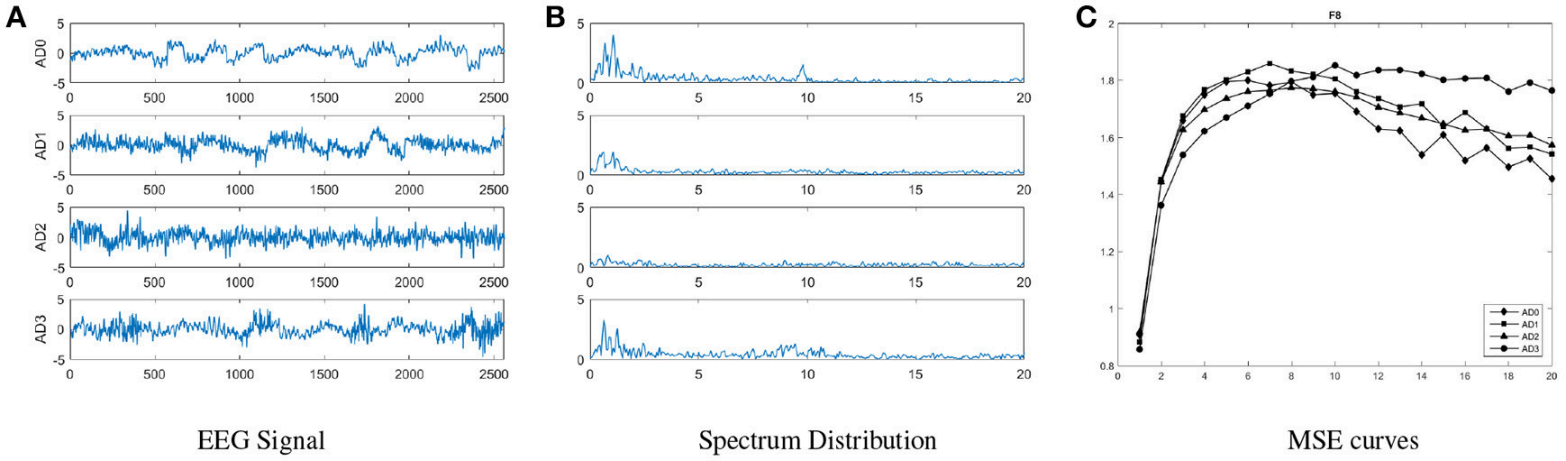
An illustration of general distribution for each group, including **(A)** raw EEG signals, **(B)** spectral powers, and **(C)** MSE on channel F8 for all groups. These curves show that MSE curves are more distinguishable than EEG signals and spectral powers in overall, and the trend with increasing scale factors in the MSE curve of each AD group is different.

In this study, we use *m* = 2 and *r* = 0.15, and the range of scale factors is [1, 20] by following our previous work (Yang et al., 2013). Figure 1 shows the averaged raw EEG signals, spectral power and MSE scores across all groups; a cross-over is observed in the MSE curves with the increasing scale factors. In short-time scales (≤ 8), lower MSE features are observed from the severe AD group comparing to normal controls, but in long-time scales (>8) an opposite pattern is observed.

### 2.4. Hybrid machine learning model for classification and biomarker identification

The objective of applying machine learning model to analyze the MSE features of EEG signals is two-fold: first, we intend to discriminate between control group and AD groups (AD1, AD2, and AD3) by performing a binary classification task in a one-to-one manner (exhaust all the possible combination of pairs). Second, we aim to examine the multivariate correlation patterns between MSE features and dementia symptoms rated by clinicians based on The Neuropsychiatric Inventory (NPI) (Cummings et al., 1994). After extracting MSE features from 19-channel EEG device using 20 scale factors, 380 (= 19 × 20) dimensions were obtained for the feature space. The machine learning model may be over-fitted in training with the relatively less samples on this high dimensional feature space. Therefore, regularization learning methods are employed to perform classification tasks between different AD/HC groups while reducing the dimensionality of trained model. A logistic regression (LR) model is trained and fitted with a penalization on the number of features with non-zero coefficients. As a result, an automatic feature selection is performed by forcing some features to yield zero coefficients. In the following subsections, we present two classic types of regularized LR models. Furthermore, we implement canonical correlation analysis, a unsupervised learning method, for inferring the correlations among two sets of variables.

#### 2.4.1. *L*_1_-norm and *L*_2_-norm regularized learning methods

The original form of LASSO is a linear regression model with a penalty term that controls the number of non-zero coefficients for all variables. In a classification problem, LASSO is reformulated with the cost function of LR, which is rewritten as the following problem (Tibshirani, 1996; Friedman et al., 2001):

(3) $$\begin{array}{c} \begin{array}{c} \underset{\beta_{0},\beta }{\max}\left\{\sum_{i=1}^{N}\left[y_{i}(\beta_{0}+\beta^{T}x_{i})-\log(1+e^{\beta_{0}+\beta^{T}x_{i}})]-\lambda \overset{p}{\underset{j=1}{\sum^{\text{​}}}}|\beta_{j}\right|\right\}, \end{array} \end{array}$$

where $f\left(x_{i}\right)=\beta_{0}+\beta^{T}x_{i}$ and *y*_*i*_ are the prediction and target class for the *ith* sample respectively. $\overset{p}{\underset{j=1}{\sum }}|\beta_{j}|$ is also known as *L*_1_-norm penalty that controls the shrinkage with corresponding parameter λ selected via nested cross-validation.

However, LASSO attempts to addresses cluster information of correlated variables, which is referred to as grouping effect. It only selects one and drops the other variables when fitted with a group of related variables (Zou and Hastie, 2005). In this study, this grouping effect is observed among MSE features extracted from the same electrode; however, we may want to keep multiple correlated MSE variables in our model in order to characterize the correlation in spatial patterns of functional brain activity. Therefore, we used ENet, a variation of LASSO, to account for this grouping effect (Zou and Hastie, 2005). Similar to Equation (3), ENet is formulated with a penalty term but in a different format:

(4) $$\begin{array}{c} \lambda \sum_{j=1}^{p}[(1-\alpha )\|\beta_{j}\|+\alpha |\beta_{j}|], \end{array}$$

where α is a trade-off parameter that controls the balance between *L*_1_-norm and *L*_2_-norm. As α approaches 1, the sparsity of solution will increase such that α = 1 is equivalent to LASSO. On the other hand, α = 0 is equivalent to ridge regression. As α approaches 0, the algorithm tends to encourage group selection of correlated features and stabilize the solution path. In our study, we choose the α = 0.7 for ENet as a empirical choice.

#### 2.4.2. CCA between MSE and cognitive declines

**Figure 2:**
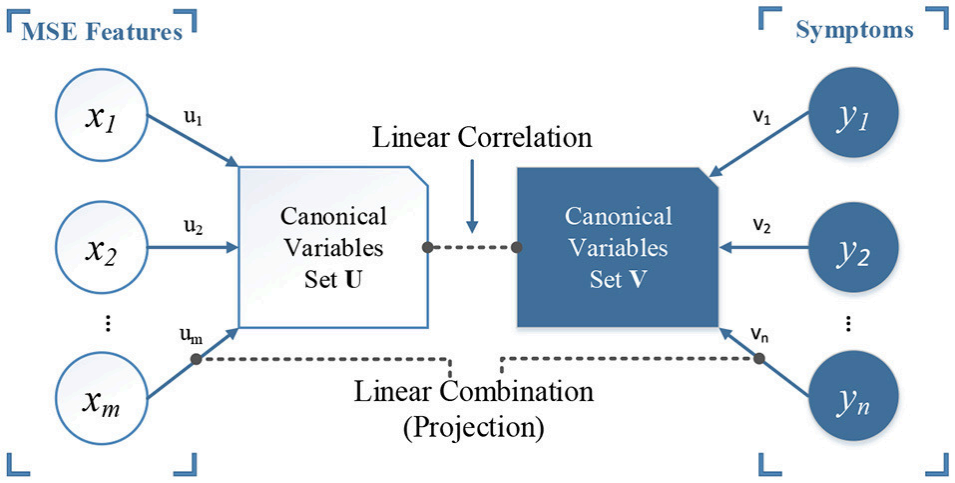
An illustration of canonical correlation analysis. The objective is to find a linear combination (projection) of set ***X*** and ***Y***, or the rotated canonical space, by maximizing the linear correlation between the two sets of new canonical variables ***U*** and ***V*** (ρ = 1 in our case). In our study, we have MSE features as set ***X*** and the scores of 12 symptoms from NPI scale as set ***Y***.

In our study, we used CCA for analyzing the multivariate correlation patterns between MSE features and cognitive decline symptoms related to dementia. The NPI scores includes 12 symptoms: delusions (DEL), hallucinations (HAL), agitation (AG), dysphoria (DEP), anxiety (ANX), apathy (APA), irritability (IRR), euphoria (EUP), disinhibition (DIS), aberrant motor behavior (ABE), night-time behavior disturbances (NIG), and appetite and eating abnormalities (APP). CCA (Hotelling, 1936) is a multivariate analysis approach for finding the relationship between two sets of variables, **X** and **Y**, with the objective to maximize the Pearson correlation based on projections on new subspaces of **X** and **Y**. Figure 2 illustrates the cencept. The new feature space is constructed by canonical variables set **U** and **V**, which correspond to original MSE and symptoms rating scales. CCA is formulated as follows:

(5) $$\begin{array}{l} \underset{u\in R^{p},v\in R^{q}}{\text{argmax}}\;\frac{u^{T}X^{T}Yv}{\sqrt{\left(u^{T}X^{T}Xu)(v^{T}Y^{T}Yv\right)}}, \end{array}$$

where **X** is a *n* × *p* matrix that represents *n* samples in *p*-dimensional space; **Y** is a *n*×*q* matrix that represents *n* samples in *q*-dimensional space; **X** and **Y** are two sets of paired variables that correspond to *n* samples. This problem is solved as a generalized eigen-decomposition problem.

#### 2.4.3. Model validation and biomarker identification

The evaluation of overall performance uses the following three metrics: (1) *accuracy* indicates the ratio of correctly classified patients in the entire sample; (2) *sensitivity* indicates the ratio of correctly identified AD patients; and (3) *specificity* indicates the ratio of correctly identified normal controls, defined as follows:

(6) $$\begin{array}{rc} \text{Accuracy}= & \;\frac{\text{TN}+\text{TP}}{\text{TN}+\text{TP}+\text{FP}+\text{FN}}, \end{array}$$

(7) $$\begin{array}{rc} \text{Sensitivity}= & \;\frac{\text{TP}}{\text{TP}+\text{FN}}, \end{array}$$

(8) $$\begin{array}{rc} \text{Specificity}= & \;\frac{\text{TN}}{\text{TN}+\text{FP}}, \end{array}$$

where TP = true positive, TN = true negative, FP = false positive, and FN = false negative. In particular, normal control group is treated as the negative class in the classification task of this study. If two groups are both AD patients, the less severe group is defined as the negative class. We reported the accuracy of both training and test set to show the potential risks of overfitting, indicated by the gap between training and testing accuracy.

The classifier will be impacted by the imbalanced data during training phase, and the trained model is usually more biased to the majority class. The Receiver Operating Characteristic (ROC) analysis is thus employed for performance evaluation. The area under ROC curve (or AUC) is used as an alternative metric without bias from the selection of threshold parameter (e.g., cut-point) in binary classification of logistic regression.

In addition, we use a leave-one-subject-out cross-validation design to minimize the bias introduced by sample variability. That is, the generalization error is estimated by leaving out samples collected from in the three sessions of one participant for testing and training the model on remaining samples. Validation repeats for all participants as testing samples. Furthermore, the importance of EEG biomarkers was assessed by overall selection frequency in all iterations.

## 3. Results

### 3.1. Classification for AD severity

Table 1 presents the classification performances of three algorithms. AD groups are considered as the target class. ENet classifier with α = 0.7 (Enet 0.7) yields the best accuracy for classification tasks of HC vs. AD2 and AD1 vs. AD2, and LASSO classifier performs better in discriminating HC vs. AD1, AD1 vs. AD3, and AD2 vs. AD3. Neither model is able to classify AD1 vs. AD2 given the low specificity, although the AUC achieved ~0.7. From the feature selection perspective, grouping effect is accounted for in ENet, which allows for multiple selection among correlated MSE features. This property, considering the high correlation among EEG biomarkers, may better describe the topological patterns for brain activity. Finally, LR with no regularization performed 100% accurate for the training tasks, but the model has poor generalizability because of low test set accuracy and AUC, which indicates the over-fitting issue. All the above results show that the regularized learning methods provide insights about EEG biomarkers with lower risks of over-fitting than LR models.

**Table 1:** Summary of classification performances for all classification tasks among three methods LASSO, Enet, and LR.

**Method**	**Group**	**Sensitivity (%)**	**Specificity (%)**	**Test accuracy (%)**	**Train accuracy (%)**	**AUC**
LASSO	HC vs. AD1	40.54	43.40	42.22	51.72	0.45
	HC vs. AD2	86.44	28.00	69.05	87.55	0.64
	HC vs. AD3	88.71	69.09	**79.49**	95.61	**0.83**
	AD1 vs. AD3	90.48	72.22	**82.05**	96.49	**0.87**
	AD2 vs. AD3	47.19	84.21	**72.40**	80.80	**0.71**
	AD1 vs. AD2	87.64	31.08	71.03	84.74	0.69
Enet (α = 0.7)	HC vs. AD1	43.90	44.90	44.44	51.72	0.47
	HC vs. AD2	86.59	28.77	**69.84**	87.15	**0.64**
	HC vs. AD3	87.30	68.52	78.63	96.49	0.83
	AD1 vs. AD3	88.89	70.37	80.34	100.00	0.86
	AD2 vs. AD3	46.67	84.13	72.04	82.25	0.71
	AD1 vs. AD2	88.20	32.43	**71.83**	86.75	**0.70**
LR	HC vs. AD1	56.10	55.10	55.56	100.00	0.54
	HC vs. AD2	83.66	20.20	58.73	100.00	0.53
	HC vs. AD3	68.85	46.43	58.12	100.00	0.61
	AD1 vs. AD3	71.64	52.00	63.25	100.00	0.64
	AD2 vs. AD3	32.00	79.22	58.06	100.00	0.56
	AD1 vs. AD2	17.53	81.94	57.14	100.00	0.49

*None of LR models has achieved 60% accuracy, so they are considered as ineffective models with no highlights. LASSO outperforms the other two methods for tasks HC vs. AD3, AD1 vs. AD3, and AD2 vs. AD3. Enet outperforms for tasks HC vs. AD2 and AD1 vs. AD2. The LR performs worst in all tasks. Bold highlights the best performances with accuracy at least 60%*.

### 3.2. Multivariate correlation between MSE and cognitive declines

**Figure 3:**
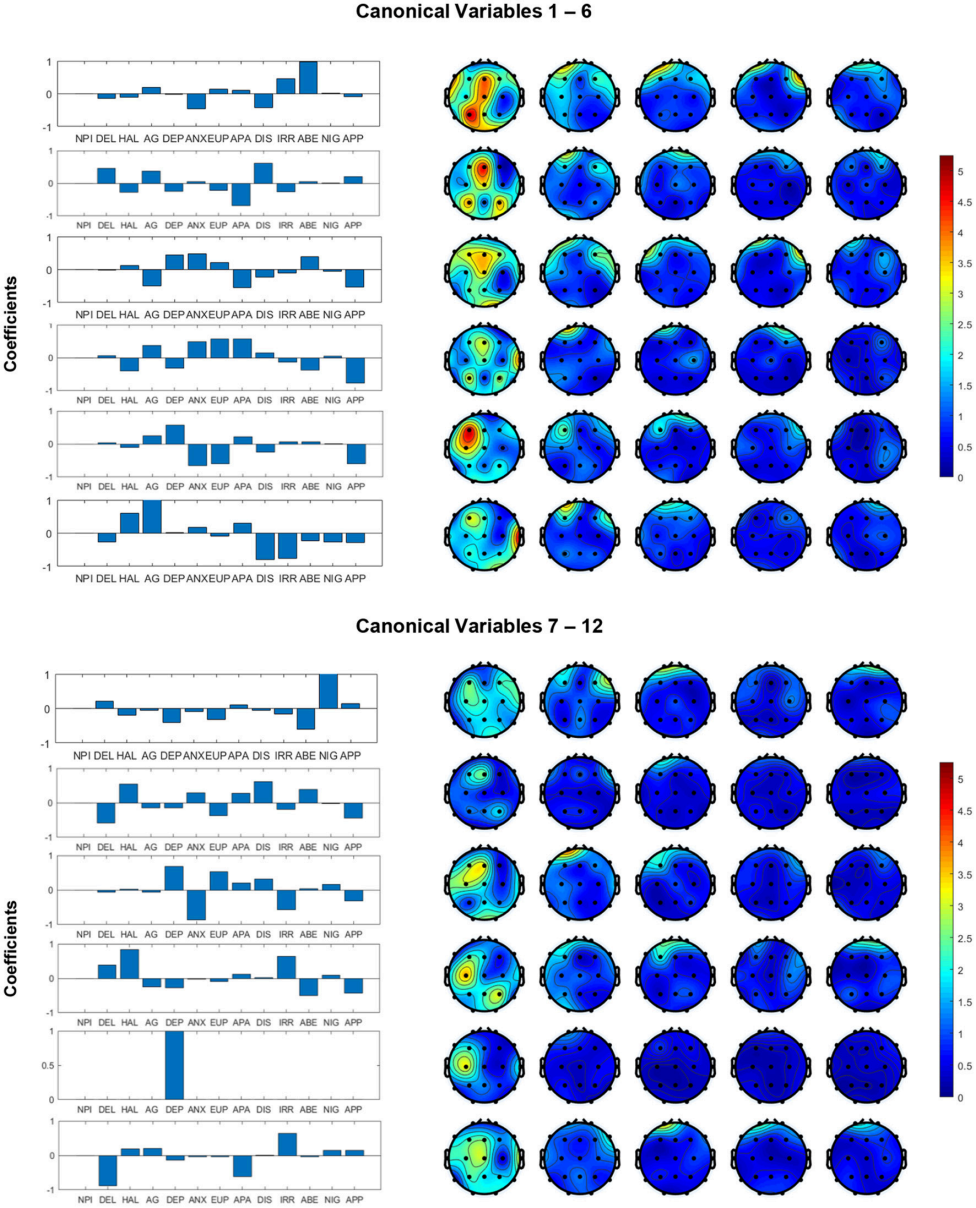
Structural coefficients of canonical variables reformed from MSE and cognitive dysfunction symptoms. We decide to focus on the first six canonical variables because they yielded higher coefficients in the MSE features.

The structure coefficients in canonical variables for all channels and symptoms are presented in Figure 3. These structure coefficients can be interpreted as the loadings of each original variables (MSE features and cognitive declines) projected into the canonical space. In Figure 3, the left panel shows the coefficients of symptoms and right panel shows the absolute values of coefficients for MSE features across all channels. These figures describe how the MSE features and cognitive symptoms contributed to all canonical variables, which suggests a multivariate correlation pattern between clinician's rating and functional brain activity. Our study focus on canonical variables 1–6, since they have higher coefficients of MSE features. For example, in canonical variable 1, the combination of symptoms IRR, DIS, ANX and ABE is associated with channels P3, O1, O2 and central electrodes in short-term complexity, but associated with the frontal area in long-term complexity. In canonical variable 2, the combination of DIS, DEL and APA is associated with central-frontal region. In canonical variable 3, the combination of symptoms DEP, ANX, AG, APA and APP with is associated with frontal region. Canonical variables 4 and 5 present a similar correlation pattern between symptoms ANX, EUP, and APP, and frontal region, but with different signs (positive and negative). Canonical variable 6 presents a positive functional correlation between temporal regions with HAL and AG, but a negative correlation with DIS and IRR. We noted that most significant coefficients are assigned to low time factors (1–4), while very few non-zero coefficients are distributed in frontal regions for higher (5–8) time factors. In addition, canonical variables 7–12 yield relatively small coefficients comparing to canonical variables 1–6.

### 3.3. Topological patterns of EEG changes associated with AD severity

**Figure 4:**
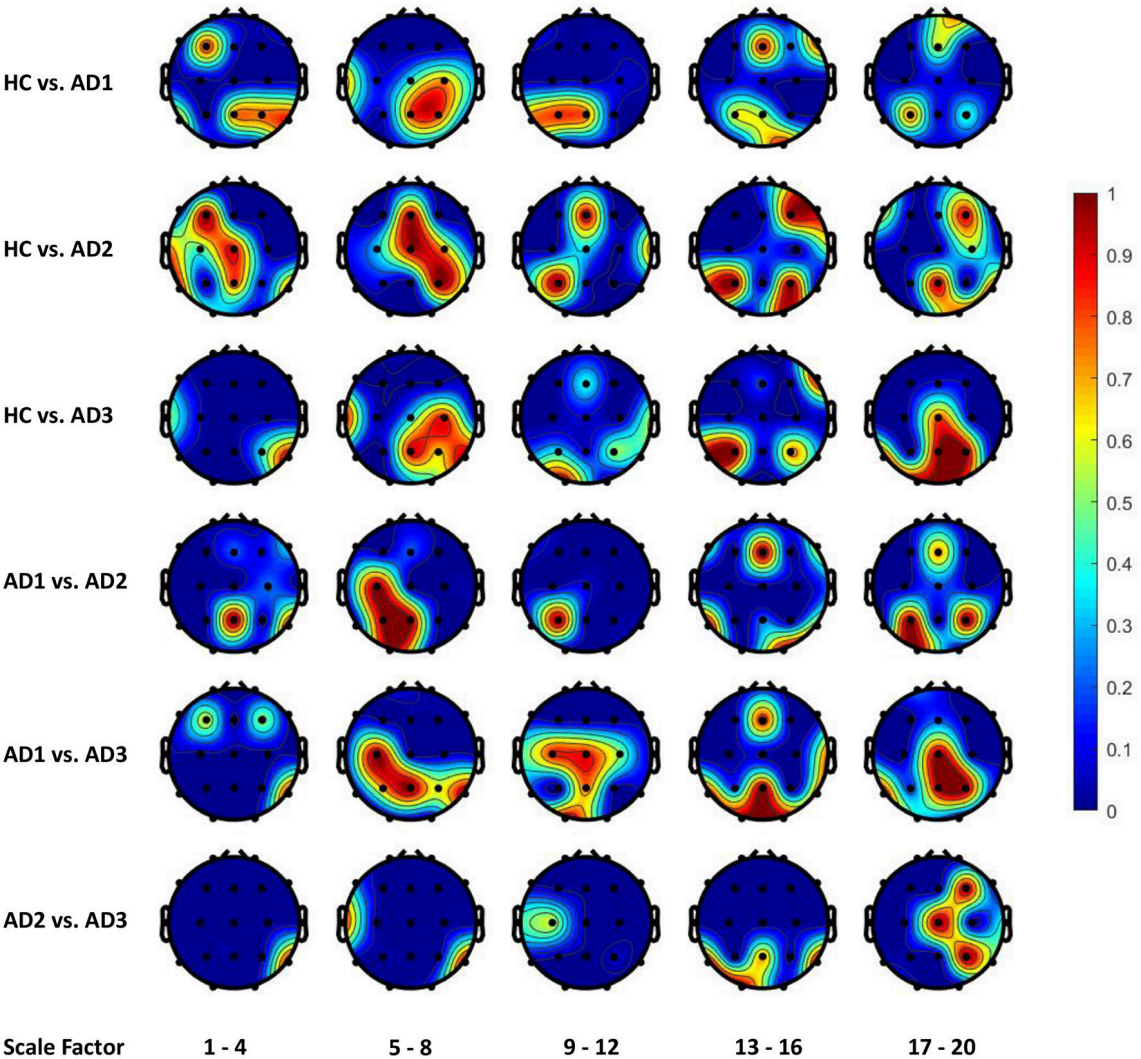
The frequency distribution of MSE features selected by LASSO across brain regions. The plotted values are the ratio of being selected in cross validation for each electrode and scale factors; we categorize the 20 scale factors (MSE features) into 5 bins; the plotted value for each bin is the maximal frequency within the bin. For instance, if scale factors 1, 2, 3, and 4 computed using channel O1 is selected in 30, 50, 70, and 90% of all replications in cross-validation, the value assigned to channel O1 will be 0.9 for the scale factors 1–4.

**Figure 5:**
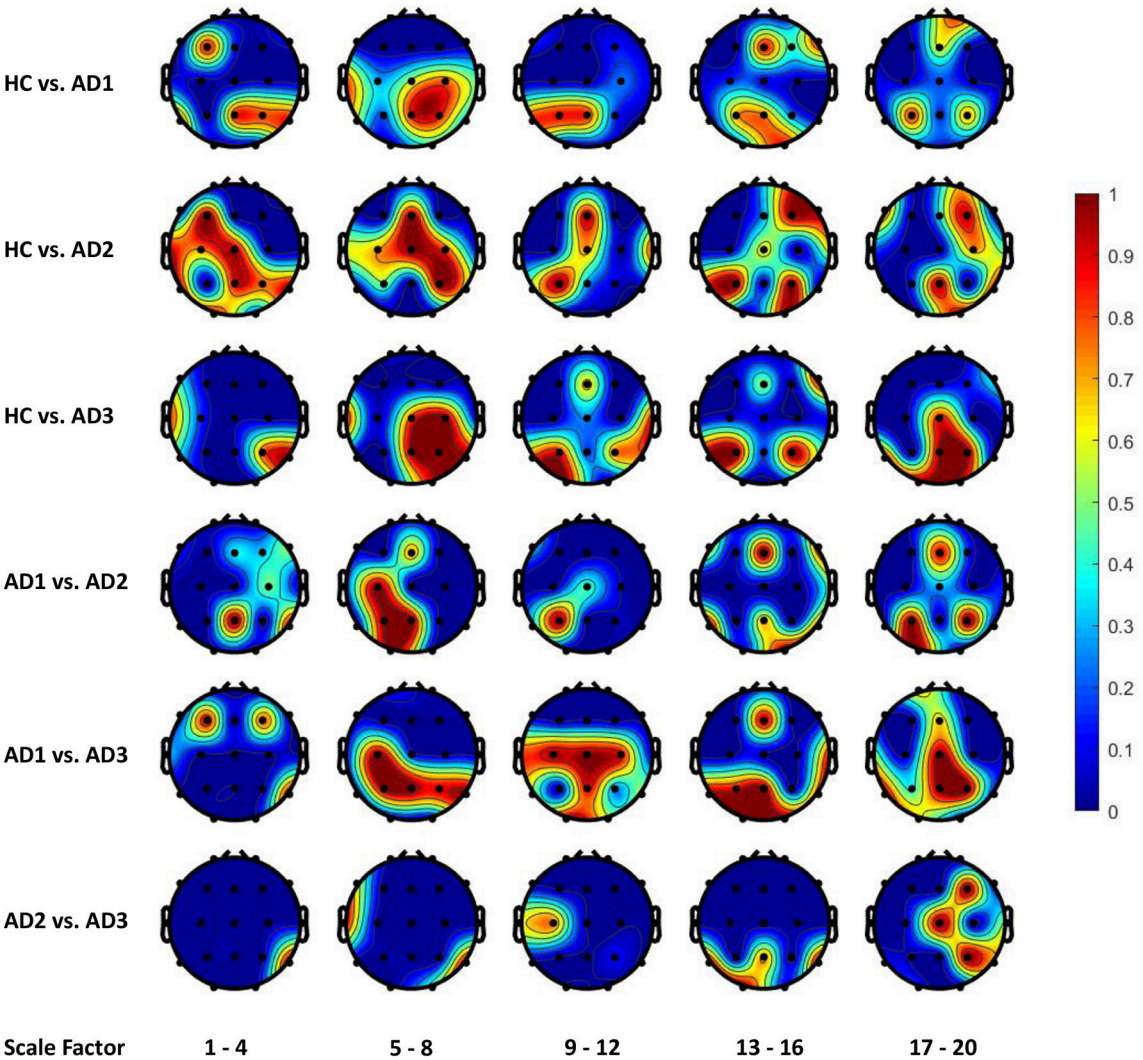
The frequency distribution of MSE features selected by Elastic Net (alpha = 0.7) across brain regions.

Figures 4, 5 display the frequency distribution of selected MSE features in all EEG channels across the brain regions. In the classification tasks of HC vs. AD1 and HC vs. AD2, the selected MSE features were concentrated in the low scale factors (1-4) and distributed diversely from frontal-central to temporal and occipital regions. In contrast, in the classification task of HC vs. AD3, a relatively consistent selection of channels was shown across subjects, mainly in channels T5, T6, O1, and O2.

## 4. Discussion

### 4.1. Classification results

In overall, we found the AD3 is most differentiable from any other groups, including both patients and controls. This result suggested a significant change in EEG complexity of moderate to severe AD patients comparing to early stage dementia. Furthermore, the mild AD patients can be discriminated from other groups in a moderate accuracy, indicating the presence of alteration in EEG dynamics can be captured (~70% accuracy). In contrast, none of our developed models can discriminate between control and very mild AD patients. However, the classification task of AD1 vs. AD3 yields the best accuracy (82.05%) using the LASSO classifier). This may imply that participants with less mild AD share very much complexity in common with healthy controls. In contrast, the classification task of HC vs. AD3 only yields accuracy = 79.49%. Although the classification performances in overall are not significantly high, our purpose is to utilize regularization methods to identify the brain activities patterns measured by nonlinear features of EEG collected from subjects including normal controls and AD cohorts at different severity levels. Limited by the inevitable data quality issues of EEG signals, the present study did not overemphasize the importance of accuracy because the models may learn false patterns as the result of achieving high performances on a noisy dataset. Instead, our study is focused on developing a robust model and providing scientific insights about a consistent pattern of EEG biomarkers across different individuals.

### 4.2. Functional activity patterns from feature selection of regularization models

From the classification task of HC vs. AD3, the LASSO classifier consistently selects MSE features from right temporal region across all folds in cross-validation. This finding may be consistent with prior studies that Alzheimer's disease is associated with rapid decline in the volume of medial temporal lobe (Jobst et al., 1994). It is possible that the atrophic changes in severe AD could result in prominent changes in functional brain activity so machine learning algorithm can consistently detect the difference between healthy elderly and patient with severe AD.

On the other hand, our results present that major changes with the progress to severe AD occur in occipital and parietal regions, in particular the right hemisphere with lower scale factors (1–4 and 5–8) and left hemisphere with higher scale factors (13–16 and 17–20). However, the classification task of HC vs. AD2 and HC vs. AD1 yields a unstable classification performances, and the selected channels are diversely distributed across different brain regions. This uncertainty may reflect the heterogeneous course of the disease observed in very early and mild AD. In other words, we should expect higher individual variability among patient from AD1 and AD2 comparing to AD3, and thus leads to a varying feature selection solution depending on the different partitioning of subsamples in cross-validation.

AD is known to have an insidious course of onset, with the functional decline leading the structural deficit during the course of illness. Previous studies of machine learning of AD focused mainly on structural brain imaging data, such as ADNI (Frisoni et al., 2010). Few studies have used functional brain activity data to classify AD. Therefore, our results may implicate in the early screening of AD in the future application using functional brain data. Our future direction may include more considerations for stabilizing the feature selection procedure across subjects during early developmental stages of AD. Variables from different sources, e.g., age, gender, spectral features, network metrics, asymmetry, synchrony patterns, can be introduced to build a more comprehensive model for classifying AD and normal control cohorts.

### 4.3. Neurological insights for AD progression

In our study, the regularization learning algorithms enable the discovery of meaningful associations between the model/feature selection and the spatial/temporal functional brain activity patterns. Specifically, in the cross-validation, we assumed the frequency of being selected for each electrode/brain region and scale factor implies how much it accounts for the between-group differentiation. Our findings suggested the posterior brain regions as the most impacted areas from cognitive declines following dementia, which is consistent with previous quantitative EEG studies (Yang et al., 2013). The electrodes picked by regularized learning algorithm in our study also have some overlap with EEG biomarkers using a multivariate extension of MSE in a recent study (Azami et al., 2017).

In addition, the multivariate correlation patterns obtained by CCA in our study suggest the grouped symptoms can provide rich information associated with MSE. We observed a collection of functional correlations of central parietal and left occipital brain regions with symptoms such as ABE and IRR, and a group of negative correlations between frontal regions with ANX, EUP, and APP. The sleep changes (reflected in NIG) were found associated with short-term complexity in occipitoparietal electrodes, which is consistent as reported by Yang et al. (2013). Our study further validated the potential of complex patterns of clustered neuropsychiatric symptoms that may be associated with EEG complexity in various regions at short- and long-term time scales.

### 4.4. Limitations and future work

The present study still has a few limitations. First, EEG data segments used in this study are relatively short (10 s), and therefore may not be able to provide long-term complexity information. Moreover, the number of trials is limited; to compromise this shortcoming, we collected multiple sessions for each participant in order to extend the sample size. Finally, since each channel was considered individually during feature extraction and classification, the interaction between electrodes may not be fully presented in our current dataset; the future work may consider connectivity patterns to give a comprehensive view of EEG alterations with AD progress. Furthermore, a multi-variate MSE (MMSE) analysis, proposed by Ahmed and Mandic (2011), that accounts for spatio-temporal dynamic brain patterns, i.e., both within-and cross-channel dependencies, will be investigated and integrated in machine learning models in our AD study.

## 5. Conclusion

In this study, we examined the functional brain activity patterns in varying AD severity levels with a contrast to normal controls. MSE was used as a measure of nonlinear dynamic to represent the signals complexity using 10 seconds of resting EEG. Regularized logistic regression was applied to this supervised machine learning problem, in which we trained leave-one-subject-out cross-validated model with the MSE features for a comparison between AD cohorts and normal controls. We demonstrated ~80% classification accuracy between severe AD cohorts and normal controls and found that the long-term complexity of EEG signals decreases with the severity of AD. Moreover, cognitive function declines can be analyzed in combination with the original MSE features to indicate the integrated correlation patterns of dementia symptoms and EEG complixity alternations. These findings relate neurological changes associated with different AD severity to the state-of-the-art assessment scales. On the other hand, regularized learning methods showed the capability for automatic selection of significant EEG biomarkers. Our future work will explore the integrative patterns including EEG complexity, synchrony and functional connectivity in this AD research direction.

## Author contributions

ACY and J-LF contributed conception and design of the study, participants recruitment, experiment conduction, and dataset collection and preprocessing. MF and C-AC are in charge of modeling and analysis methods. All authors contributed to manuscript writing and proofreading.

### Conflict of interest statement

The authors declare that the research was conducted in the absence of any commercial or financial relationships that could be construed as a potential conflict of interest. The Reviewer FZ and the handling editor declared their shared affiliation.
